# Advances in Conductive Biomaterials for Cardiac Tissue Engineering: Design, Fabrication, and Functional Integration

**DOI:** 10.3390/polym17050620

**Published:** 2025-02-26

**Authors:** Tabrej Khan, Gayathri Vadivel, Kalaivani Ayyasamy, Gowtham Murugesan, Tamer A. Sebaey

**Affiliations:** 1 Department of Engineering Management, College of Engineering, Prince Sultan University, Riyadh 12435, Saudi Arabia; tsebaey@psu.edu.sa; 2 Department of Physics, KPR Institute of Engineering and Technology, Coimbatore 641 407, Tamil Nadu, India; e.kalaivvani@gmail.com; 3 Department of Physics, Kongunadu Arts and Science College, Coimbatore 641 029, Tamil Nadu, India; gowthampsix@gmail.com; 4 Department of Mechanical Design and Production Engineering, Faculty of Engineering, Zagazig University, Zagazig 44519, Sharkia, Egypt

**Keywords:** biomaterials, cardiac tissue, functional integration, heart failure, design and fabrication

## Abstract

Heart failure functions as one of the leading global causes of death because it falls under the cardiovascular disease categories. Cardiac tissue engineering advances by developing new tissues to rebuild heart functions in individuals with damaged heart structures as it gives medical treatment possibilities to patients reaching their final stage. Most of the heart tissue consists of cardiomyocytes which make up between 80 to 90 percent of the total organ space. The cardiomyocytes retain their specialized cell structure which includes elongation, but they align to produce contractions as they span into length. After myocardial infarction, doctors need elastic soft platforms to heal the heart tissue because they mimic its natural attributes. Special consideration must be paid to the material selection for appropriate mechanical properties, given that different substances have separate qualities. Stem cell survival becomes higher, and cell differentiation develops more efficiently when a proper scaffold design is implemented, thus enabling tissue repair. Conductive biomaterials demonstrate the best candidate status for cardiac tissue engineering due to their ability to both convey electrical signals and boost biological actions as well as promote cellular communication. Scientists conduct life science research on stem cells because the cells present unique characteristics. Biomaterials with conductive properties within cardiac tissue engineering help the body recover heart tissue while improving the functionality of damaged structures in the myocardium. This article analyzes various conductive biomaterials used in biomedical practices for cardiac tissue healing applications.

## 1. Introduction

Heart transplantation remains a viable and effective treatment option despite several limitations, as cardiovascular disorders are the primary cause of heart failure and mortality globally. Cardiac tissue engineering, which uses biodegradable polymer-made cardiac patches, injectable gels, and implanted artificial blood arteries, is seen to be a potential approach to solving this problem. Natural and synthetic polymers are the two categories of biodegradable polymers. On the other hand, non-renewable petroleum resources are used by humans to produce synthetic polymers [1,2]. In cardiac tissue engineering, natural and synthetic polymers each offer unique advantages and limitations. To harness the strengths of both types, natural/synthetic composites are often used for various cardiac tissue applications. Although natural biodegradable polymers are abundant, renewable, and environmentally friendly, they have notable drawbacks, such as insufficient mechanical properties for cardiac tissue engineering, rapid degradation, limited electrical conductivity, and potential immunological responses. Synthetic biodegradable polymers have several advantages, including excellent mechanical capabilities, well-regulated structures, great processing flexibility, and few immunological concerns. However, they also have certain disadvantages, such as a lack of cell attachment and possibly poor biocompatibility. The combination of natural and synthetic materials in some applications has resulted in the creation of exciting new composite materials. Polymer nanocomposites and nanostructured polymers, known for their enhanced surface, electrical, and mechanical properties, have transformed cardiac tissue engineering by promoting tissue regeneration. This review examines recent advancements in using synthetic and natural biodegradable nanocomposite polymers for cardiac tissue engineering and discusses the potential future directions in this evolving field. The elastomeric properties of biodegradable polymers, which support tissue contraction similar to cardiac activity, have attracted significant interest in cardiovascular tissue engineering. Their excellent porosity, biocompatibility, and customizable degradation rates further enhance their suitability for tissue regeneration. These scaffolds are capable of absorbing cells, growth factors, and other substances, breaking down over time into biocompatible products while maintaining mechanical strength during tissue formation [3,4,5,6].

One of the key characteristics of cardiac physiology is excitability, which triggers the excitation-contraction coupling cycle and enables the heart’s blood-pumping function. Many heart conditions result in electrical activity blockages of any kind or are the cause of excitability deficiencies [7]. These electrical conductivity inhibitions may be brought on by remodeling cardiomyocytes or the formation of fibrotic tissues, which would impair the heart’s capacity to function in line with the body’s necessities. One method of healing a damaged heart is to combine tissue-engineering techniques with cell-based or cell-free therapies. This technique offers a promising technological advancement through the creation of three-dimensional biomimetic scaffolds [8,9]. Since cardiac muscle is an electrically conductive tissue, conductive materials have been produced for cardiac tissue engineering applications in an effort to emulate this natural characteristics.

The development of heart muscle microtissues in the direction of in vitro cardiac disease modelling and drug screening has been greatly aided by the advent of electroconductive materials. However, due to difficulties mostly with their mechanical, biodegradable, and biocompatibility, the possibility for their clinical therapeutic applications is still in the preclinical research stage [10]. Several methods have been used in the creation of cardiac micro tissue to increase the engineered constructions’ electrical conductivity, which in turn makes it easier for neighboring cells inside the scaffolds to electrically couple with one another [11,12,13]. The first strategy involves the construction of engineered cardiac tissues (ECTs) and the development of cardiac patches embedded with conductive nanomaterials. These patches are fabricated in vitro and are designed to be transplanted onto the surface of the infarcted myocardium. Their goal is to restore heart function by achieving structural and functional integration with the host tissue.The second strategy utilizes injectable conductive nanomaterials, which are directly introduced into the damaged myocardial region to support functional recovery. Together, these approaches underscore the potential of conductive nanomaterials in advancing cardiac repair and improving outcomes for patients with heart disease [14]. Engineered cardiac tissues (ECTs), constructed using biomaterials and cells, have thus become a focal point of research in recent years. Researchers have high expectations for their potential to serve as an effective treatment for myocardial infarction and other cardiac disorders.

Biomaterials encompass a wide range of materials, including ceramics, metals, composites, and polymers, which vary in size from macro to micro and nano scales. These substances can be utilized to replace or mend damaged tissues in living creatures [15]. They serve as scaffolding for cells’ continuous division and proliferation and are crucial for anchoring cells. They can be employed in tissue engineering in various ways, such as carriers, hydrogels, and scaffolds [16]. Biomaterial form and function should closely mimic the natural properties of the cardiac extracellular matrix (ECM) to support cardiac tissue engineering. It should also provide an appropriate microenvironment to improve cell survival [17,18]. In particular, biomaterials intended to facilitate cardiac repair ought to be both biocompatible and biodegradable, possess characteristics of a native myocardium in terms of both mechanical and biological aspects, facilitate cell integration, lessen the antagonistic nature of the surrounding milieu, guarantee a gradual release of bioactive molecules, and offer a suitable electrical conductivity [15,17,19].Furthermore, the incorporation of antioxidant compounds into biomaterials may act as a mediator between fibrosis and inflammation. By eliminating reactive oxygen species, antioxidant structures can reduce oxidative damage, the fibrotic response to heart repair, and the pro-inflammatory polarization of macrophages [20].

Although finding an appropriate biomaterial can be challenging, there are a number of natural and synthetic polymers and their derivatives that have been shown to resemble a number of important ECM properties [15,16,18,21].Biocompatibility, adequate mechanical qualities, hydrogel formation ability, appropriate chemistry, and a satisfactory degradation rate are some of their advantageous attributes [18,22,23]. Materials that are easily processed can be further modified to suit the specific form and properties required for cardiac tissue engineering applications [16]. Furthermore, biomaterials can serve as a platform for culturing or incorporating target stem and progenitor cells [15]. In this way, biomaterials and cells work together to promote tissue regeneration and repair, providing essential support for cellular functions like adhesion, proliferation, and differentiation [16,23]. To maximize their regenerative potential, biomaterials can also be integrated with pro-survival factors to shield transplanted cells from the hostile conditions of post-MI damaged tissues and regeneration factors that stimulate healing in the infracted heart [18].

Additionally, the tailored 3D architecture of biomaterials enhances cellular adhesion, stimulates ECM secretion, and promotes revascularization and paracrine signaling, which helps facilitate nutrient and gas transport as well as the formation of vascular substructures. Due to the heart’s contractile movement, various mechanical stresses create non-uniform 3D deformations in cardiac tissues [24]. Biomaterials designed for cardiac regeneration must withstand mechanical strain, shear forces, and shear stresses to accommodate the stretching and compression of the myocardium. Additionally, these materials should possess electrical conductivity to support the heart’s dynamic tissue functions [19,25].

## 2. Biodegradable Polymers for Cardiac Tissue Regeneration

Biodegradable polymers for cardiac tissue engineering can be developed by combining natural and synthetic polymers [1]. This combination offers unique advantages and challenges when designing heart tissue. As a result, composite materials made from natural and synthetic polymers are often recommended for specific cardiac tissue applications, leveraging the strengths of both types [3]. This section will review recent advancements in biodegradable synthetic and natural polymers in the field of cardiac tissue engineering.

### 2.1. Natural Polymers

Natural polymers are classified as nanomaterials since they are composed of nanostructured proteins and are derived from both plants and animals [26]. Natural polymers are widely used in various tissue engineering applications due to their biodegradability, renewability, and availability [27]. Collagen and chitosan are examples of natural biodegradable polymers employed in cardiac tissue engineering, as highlighted in this section [28]. Table 1 outlines some common natural biodegradable polymers and their associated benefits and limitations.

#### 2.1.1. Collagen

For cardiac tissue engineering efforts, collagen offers benefits such as strong cellular activity, biocompatibility, and thermal reversibility. There are twenty-eight different forms of collagen that have been found in human tissues, including skin, tendons, ligaments, and cartilage [45]. Collagen types 1, 2, 3, and 4 are frequently investigated in the field of tissue engineering. Type 1 collagen is the most extensively utilized due to its excellent biocompatibility, making it ideal for various tissue engineering applications [46,47,48,49,50,51,52]. As a biodegradable polymer, collagen is highly valued across multiple tissue engineering domains, particularly in cardiac applications, because of its low toxicity, non-allergenic properties, biodegradability, and strong biocompatibility [32,53].

In cardiac tissue engineering, collagen scaffolds, particularly in nanofibrous forms, have been studied for their potential. Punnoose et al. describe a simple modern technique for creating collagen-based nanofibrous scaffolds that involves using an electric field to stretch a polymer solution toward a grounded collector. Type 1 collagen, often the preferred choice for these biomaterials, was used to produce nanofibrous scaffolds through fluoroalcohol-assisted methods. There have been numerous studies conducted in an attempt to find a cost-effective and nontoxic solvent because fluoroalcohols are costly and caustic. This solvent proved inexpensive and preserved the original integrity of the electrospun collagen [53]. Joanne et al. looked into electrospun collagen scaffolds in another study using cross-linking agents (glycerol) and biologically acceptable solvents (ethanol, water, and salts) [54].

Collagen scaffolds face challenges such as a limited mechanical strength, low physical durability, and reduced structural stability when hydrated. To enhance the mechanical properties of collagen-based scaffolds, a physical and chemical cross-linking of collagen fibers is often employed. For example, Lin et al. investigated 3D collagen type I scaffolds with controlled stiffness to facilitate mesenchymal stem cell differentiation and proliferation into cardiac progenitor cells. By using varying ratios of the cross-linking agents hydroxysuccinimide (NHS) and 1-ethyl-3-(3-dimethyl amino propyl) carbodiimide (EDC), the researchers optimized the stiffness of the collagen scaffolds. Their results demonstrated that a 50/50 EDC/NHS ratio yielded scaffolds with an enhanced interconnectivity and an increased Young’s modulus. Furthermore, mesenchymal stem cells cultured on these stiffness-controlled scaffolds successfully proliferated and differentiated into cardiac progenitor cells, even in the absence of TGF-β2 [32].

**Figure 1:**
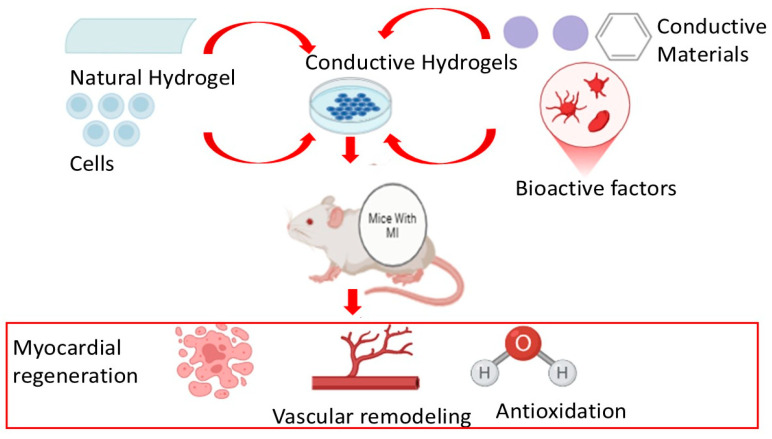
A schematic representation of the conductive hydrogel integration for cardiac repair.

Collagen scaffolds can also be made stronger mechanically by mixing them with natural synthetic polymers and inorganic components (Figure 1) [31].

Figure 1 illustrates the formation and application of conductive hydrogels combining natural hydrogels, conductive materials, cells, and bioactive factors. The integration of these components facilitates the development of conductive hydrogels for myocardial regeneration. Conductive hydrogels, injected into an in vivo model, promote myocardial regeneration, vascular remodeling, and antioxidation. The figure emphasizes the synergy between conductive materials and bioactive factors, enhancing their therapeutic potential for cardiac tissue engineering. The experimental conditions highlight the focus on in vivo applications to study myocardial repair mechanisms. In some studies, collagen has been blended with carbon nanotubes (CNTs) to enhance its mechanical and electrical properties. This approach leverages CNTs’ exceptional mechanical strength, electrical conductivity, and high aspect ratio to strengthen the overall performance of the collagen-based material [55,56]. In one investigation, Sun and colleagues synthesized collagen hydrogels with CNT integration and assessed the proliferation and behavior of cardiomyocytes within the hydrogels. The findings demonstrated that adding different weight percentages of CNTs to collagen hydrogels increased their respective elastic moduli values. Additionally, the hydrogel conductivity was improved when compared to pure collagen hydrogels, and the assembly of neonatal cardiomyocytes and cell–cell alignmentswere improved. The addition of CNTs (up to 1 weight percent) improved the cell adhesion and elongation while showing minimal toxicity to cardiomyocytes [55].

Similar work was done by Yu et al. In this study, type I collagen hydrogels at different concentrations of about 4 mg/mL and 2 mg/mL were mixed with different weight percentages of CNTs to create CNT-collagen hydrogels. The findings indicated that collagen fibers coated the surfaces of carbon nanotubes (CNTs) and that the CNTs were uniformly distributed throughout the CNT-collagen hydrogels. Remarkably, the addition of CNTs did not alter the fiber thickness or pore size when compared to pure collagen hydrogels. LX-2 cells were seeded onto the collagen, with or without CNT integration, and after three days of culture, the presence of CNTs in the hydrogels showed no significant morphological changes or signs of cell death. Additionally, CNT-collagen hydrogels demonstrated improved cardiomyocyte function compared to pure collagen hydrogels. The inclusion of CNTs in type I collagen hydrogels enhanced both the mechanical strength and electrical conductivity of the scaffolds while maintaining their original sub-micron fiber structure [56].

Recent research has also examined collagen-based cardiac patches withelectroconductive properties for myocardial infarction (MI) therapy. For instance, Hosoyama et al. created nanoengineered cardiac patches by integrating silver (Ag NPs) and gold nanoparticles (AuNPs) into collagen, which provided electroconductive properties beneficial for cardiac tissue repair. These patches featured aligned collagen fibers within an elastic hydrogel matrix. In a mouse model of MI, the patches with AuNPs and Ag NPs were evaluated for their ability to enhance electrical and mechanical repair.

The results demonstrated that in an in vivo myocardial infarction model with an established scar, only patches incorporating AuNPs significantly enhanced connexin-43 expression in newborn rat cardiomyocytes subjected to electrical stimulation, leading to improved cardiac function. The fibers with incorporated AuNPs exhibited a stable electrical conductivity under physiological conditions. In contrast, the Ag NP fibers demonstrated a reduced stability due to accelerated nanoparticle degradation. Thus, AuNPs containing patches show promise as a therapeutic option for MI, although stability considerations may still be necessary [57].

#### 2.1.2. Chitosan

Tissue engineering has increasingly focused on chitosan, a linear polysaccharide derived from chitin through partial deacetylation, due to its structural similarity to natural glycosaminoglycans. This similarity contributes to its low toxicity and excellent biocompatibility [58,59]. In vivo, chitosan breaks down through enzymatic hydrolysis into non-toxic byproducts, and its ability to support cell recognition and cytocompatibility makes it valuable for tissue engineering. Chitosan and its derivatives are frequently coated or grafted onto scaffolds; however, its limited solubility at the physiological pH presents a challenge [44,60,61,62,63].

Recently, chitosan-based hydrogels have been explored in cardiac tissue engineering as biodegradable materials with controllable degradation properties. For example, Xu et al. developed a temperature-responsive chitosan hydrogel as an injectable scaffold for mesenchymal stem cell (MSC) delivery. Their study demonstrated that this hydrogel enhanced the graft size, improved MSC retention in ischemic heart tissue, and promoted MSC-driven neovascularization and cardiomyocyte differentiation. Additionally, the hydrogel contributed to improved cardiac function and hemodynamics in the infarcted region of rats five weeks post-myocardial infarction [62].

Chitosan hydrogels have also demonstrated their potential as scaffolds for cardiovascular applications. Aussel et al., for example, investigated these hydrogels for small-diameter vascular grafts. Both in vivo studies on rats and sheep and in vitro tests demonstrated a good hemocompatibility and promising biocompatibility for chitosan-based hydrogels [64].

To promote an effective regeneration of infarcted heart tissue, it is essential to enhance the electrical properties of grafts. Research has shown that incorporating gold nanoparticles (AuNPs) into chitosan scaffolds improves the electrical connectivity between adjacent cells. In one study, Baei et al. developed injectable hydrogels that were thermosensitive and electroconductive by embedding AuNPs into chitosan for cardiac use. By adjusting the AuNP concentration within the hydrogel, the team could regulate its gelation and conductivity, finding that a 1% AuNP concentration provided a conductivity comparable to native myocardial tissue (0.13 S/m). This AuNP-chitosan hydrogel also enhanced the differentiation of mesenchymal stem cells (MSCs) into cardiac lineages and supported MSC viability and growth [9].

While AuNPs show promise in cardiac applications, their toxicity poses a challenge. Selenium nanoparticles (SeNPs), which are naturally found in the body, have emerged as an alternative due to their therapeutic potential in cardiovascular disease, as low selenium levels are linked to heart disease. In 2018, Kalishwaralal et al. created a composite film of chitosan and selenium nanoparticles (SeNPs) that achieved a high electrical conductivity (0.0055 S/cm) and a tensile strength of 419 kPa, comparable to that of native heart tissue. This composite effectively supported cell growth and proliferation, with cells forming membrane nanotubes that indicated an enhanced transmission of electrical signals, increased cardiac cell activity, and improved cardiac repair potential. Additionally, the study noted that the rough surface texture of the nanofibers helped reduce the inflammation risk, emphasizing the importance of nanofiber morphology and surface characteristics in lowering immune responses [65,66,67,68].

Despite their advantages, natural polymers like chitosan still face challenges in cardiac tissue engineering, including a rapid degradation, limited electrical conductivity, immunogenicity, and insufficient mechanical strength [69].

### 2.2. Synthetic Polymers

Synthetic, biodegradable polymers, including polylactide (PLA), poly(glycolic acid) (PGA), polylactide-glycolic acid (PLGA), polyethylene glycol (PEG), polyurethane, polycaprolactone (PCL), and poly(N-isopropylacrylamide), have been widely utilized in heart tissue engineering [70]. These polymers are particularly attractive for cardiac applications due to their favorable physical and chemical characteristics, such as their robust mechanical strength, customizable structures, excellent processing versatility, and low immunogenicity. These properties make them suitable for engineering cardiac muscle tissue. The following sections will explore the commonly used biodegradable synthetic polymers (Table 2) in cardiac tissue engineering.

#### 2.2.1. Polycaprolactone

ε-caprolactone undergoes ring-opening polymerization to form polycaprolactone (PCL), a synthetic biodegradable polymer. PCL is commonly chosen for tissue engineering applications due to its outstanding mechanical properties, flexibility, durability, and excellent biocompatibility [75]. It is particularly suitable for applications requiring a slow degradation rate, with a degradation time of approximately 2–3 years. However, this long degradation period presents a challenge in cardiac tissue engineering applications, where a faster resorption is often needed [76,77,78,79].

To enhance PCL’s mechanical and degradation properties, two main strategies are employed as follows: incorporating nanostructured fillers and using PCL as a copolymer or component in blended materials [80]. For instance, Ghaziof et al. developed PCL/multiwalled carbon nanotube (MWCNT) composite scaffolds with varying MWCNT concentrations using solvent casting and vacuum drying techniques for myocardial tissue engineering. Their findings showed that incorporating MWCNTs enhanced both the mechanical strength and electrical conductivity of the scaffolds. Specifically, the elastic modulus increased by 85%, 45%, and 45% for MWCNT concentrations of 1, 0.75, and 0.5 wt.%, respectively. Additionally, the electrical conductivity was improved to 11.32, 8.15, and 5.23 μS/cm at these concentrations [81].

In another study, Castilho et al. developed scaffolds for cardiac applications by utilizing melt electrospinning to create poly(hydroxymethylglycolide-co-ε-caprolactone) (pHMGCL) scaffolds. The pHMGCL scaffolds demonstrated improved cellular responses due to the mechanical anisotropy created during melt electrospinning, as well as an enhanced cell alignment compared to typical PCL scaffolds [82].

To further improve the functionality for engineered cardiac tissue, conductive polypyrrole was combined with PCL. Spearman et al. explored a conductive polypyrrole–PCL interpenetrating network as an electroactive substrate for cardiomyocyte cultures. Their results showed that polypyrrole–PCL films facilitated cardiomyocyte attachment, reduced cell size, and decreased surface hydrophobicity. Additionally, cardiomyocytes cultured on these films exhibited an improved peripheral localization of the gap junction protein Cx43 (60.3% on polypyrrole–PCL vs. 46.6% on sodium hydroxide-treated PCL). HL-1 cardiomyocytes grown on polypyrrole–PCL films also displayed shorter calcium transient durations (910 ± 63 ms) and faster calcium transient velocities (1612 ± 143 μm/s) compared to those cultured on control PCL films [83].

Furthermore, various metallic and non-metallic nanoparticles, such as silver, chitosan, titanium oxide, silica, and magnetite, have been incorporated into PCL scaffolds to enhance their physicochemical properties, thereby expanding PCL’s potential in cardiac tissue engineering applications [84,85,86,87,88,89].

#### 2.2.2. Polyurethanes

Polyurethanes (PUs), a type of synthetic polymer, are commonly used in medical applications due to their excellent biocompatibility, mechanical strength, stability, and durability. These properties make PUs ideal for devices such as artificial hearts, bladders, heart-assist balloon pumps, and vascular grafts. However, the long degradation time of PUs limits their application in cardiac tissue engineering [90].

Recent research has focused on enhancing PU scaffolds for cardiovascular applications by incorporating nanostructured fillers. For example, Nazari et al. employed electrospinning to fabricate graphene-silver/polyurethane (rGO-Ag/PU) nanofibrous scaffolds for cardiac tissue engineering. The incorporation of rGO-Ag enhanced the electrical conductivity, tensile strength, and wettability of the PU nanofibers. These biocompatible scaffolds improved the cell adhesion and facilitated the cardiogenic differentiation of human cardiac progenitor cells (hCPCs) in vitro, all without the need for cardiogenic additives in the culture media [91,92].

In a separate study, Shokraei et al. created carbon nanotube (CNT)/PU nanofibrous scaffolds using a hybrid electro-spray/spinning method. The addition of CNTs notably improved the electrical conductivity, Young’s modulus, tensile strength, and hydrophilicity of the CNT/PU nanocomposites while also reducing the fiber diameter. This approach produced scaffolds with enhanced mechanical properties and conductivity, even at low CNT concentrations. The scaffolds also promoted a high cell attachment, viability, and proliferation of cardiomyoblasts, indicating their potential for effective cardiac tissue repair [93].

## 3. Conducting Polymers: Enhancing Cardiac Tissue Engineering

Due to their exceptional ability to repair and enhance the electrical functionality of damaged myocardial tissues, conductive polymers have become essential components of cardiac tissue engineering. Among these, polypyrrole (PPy) and polyaniline (PANI) have gained significant attention for their electrical conductivity, biocompatibility, and ability to support cellular functions [94]. PPy is widely recognized for its excellent conductivity and capacity to mimic the electrophysiological environment of cardiac tissues, though its mechanical limitations necessitate composite integration. Similarly, PANI offers a high conductivity and robust synthesis processes, making it suitable for cardiac scaffolds, but its inherent stiffness and brittleness often require modification to enhance mechanical properties [95]. The widespread use of these polymers in cardiac repair is welldocumented, with numerous studies highlighting their role in promoting cell adhesion, proliferation, and electrical stimulation. Their relevance is further underscored by the availability of extensive supporting literature, emphasizing their potential to develop effective cardiac scaffolds and devices for myocardial regeneration [96]. Conductive polymers (CPs) have garnered substantial interest as biomaterials due to their natural conductivity, relative flexibility compared to traditional conductors like metals, and straightforward synthesis processes. Adjusting synthesis parameters enables a precise tuning of CPs’ physical properties, which is valuable for specific tissue engineering applications. Polypyrrole (PPy), a notable CP, holds potential in tissue engineering, though research has primarily focused on neural and muscle cells rather than cardiac applications. Notably, PPy has shown a compatibility with cardiac cells, enhancing conductive benefits. For example, Nishizawa et al. developed PPy-coated microelectrodes to electrically stimulate cardiac myocytes, leading to synchronized contractions. Additionally, PPyhas been formed into fibrous 3D scaffolds that facilitate a higher cardiomyocyte differentiation compared to standard tissue culture surfaces [97,98,99,100,101,102,103,104,105,106,107,108,109,110,111,112,113,114].

In cardiac tissue engineering, PPy’s success has been linked to its structural properties, which affect cell support. PPy (doped with various agents) creates a non-toxic surface conducive to cell adhesion and growth, with CPs showing a responsiveness to physical texture. Future research aims to further explore how synthesis adjustments impact both the dopant’s effects and the polymer’s physical properties. CPs offer distinct advantages for engineering electrically conductive tissues like cardiac muscle, with recent studies demonstrating their usefulness in biosensors, drug delivery systems, and scaffold applications. CPs’ unique conjugated backbones facilitate electron exchange, creating an environment that encourages stem cells to differentiate into electroactive tissues. Moreover, CPs have been shown to mediate cell adhesion via integrin and non-integrin interactions with proteins on their surfaces. However, challenges remain, including CPs’ limited mechanical properties, processability, and biocompatibility, as well as concerns over electrical degradation over time. To address these, researchers are focusing on CP composites with biocompatible polymers and investigating ways to immobilize dopants to enhance electrical stability [115,116,117,118,119,120,121,122,123].

Among CPs, polyaniline (PANI) is a popular choice for cardiac applications due to its favorable biocompatibility, ease of synthesis, and stability. PANI has been blended with biodegradable polymers to develop composites with suitable mechanical and electrical properties. In studies, PANI-based nanofibers improved cell proliferation and alignment, maintaining cell morphology for cardiac tissue engineering. Another investigation with PANI-PLA nanofibrous sheets showed an enhanced differentiation in cardiomyoblast cells. This blend, with random and aligned fibers, provides an ECM-like environment that supports cell attachment and promotes angiogenesis, mimicking native cardiac tissue properties [124,125].

For cardiac applications, our team developed an electroactive polyurethane (AP-PU) containing an aniline pentamer. Blending AP-PU with polycaprolactone (PCL) provided favorable electrical properties and antioxidant potential, helpful in oxidative-stress-affected tissues. Additional efforts have focused on electroactive AP-PU/PCL scaffolds, which have supported cardiac cell adhesion and gene expression effectively, and PU/siloxane films with an aniline tetramer, which have shown compatibility with cardiac cells [126]. Qazi et al. explored PANI-PGS composites, finding significant improvements in cell proliferation, with PANI helping to buffer pH and mitigate acidic byproducts [127].

Polypyrrole (PPy) is also widely used for cardiac engineering because of its high conductivity and biocompatibility. While PPy’s inherent brittleness can be limiting, composites with materials like silk fibroin and PCL have enhanced their flexibility and bioactivity [128,129]. For instance, PPy-PCL scaffolds provided a conductive environment for a cardiomyocyte culture, improving cell adhesion and signaling properties, suggesting PPy-PCL could serve as a viable conductive material for cardiac tissue applications [83].

Another widely researched CP, poly(3,4-ethylenedioxythiophene) (PEDOT), is known for its conductivity and environmental stability. PEDOT: PSS, a biocompatible form of PEDOT, has demonstrated a significant conductivity and transparency, making it suitable for cardiac applications [130,131]. Roshanbinfar et al. incorporated PEDOT: PSS into collagen and alginate hydrogels to develop a composite that improved conductivity and promoted alignment and maturation in cardiac cells, showing a potential for enhancing electrophysiological properties in cardiac tissues [132].

Due to their ability to influence cell behaviors such as adhesion, proliferation, and differentiation, conductive polymers (CPs) and their composites hold promise for applications in cardiac tissue engineering. CPs can mimic native tissue signaling pathways, potentially aiding cardiac cell maturation through localized conductive surfaces. While CPs provide benefits for electrical signaling, further research is needed to assess in vivo biocompatibility, long-term electrical stability, and cytotoxicity. Additional studies should also address CPs’ brittleness and processability challenges while clarifying how CPs regulate cardiac marker expression [133].

### A Specification of the Testing Conditions for Conductivity Measurements

An accurate measurement of electrical conductivity is critical for evaluating the performance of conductive biomaterials in cardiac tissue engineering. Reported values can vary significantly depending on the testing conditions and methods employed. Common techniques include the four-point probe method, which provides precise surface conductivity measurements, and impedance spectroscopy, widely used for analyzing bulk conductivity under physiological conditions. Environmental factors such as temperature and humidity can significantly influence conductivity values, as hydrated states often mimic in vivo conditions more closely. Additionally, the adoption of testing standards, such as ASTM or ISO protocols, ensures consistency and comparability across studies. For example, a study on graphene-based cardiac scaffolds reported conductivity values of 10–100 S/cm under hydrated conditions at 37 °C using impedance spectroscopy [134]. Providing these methodological details enables a more accurate interpretation of conductivity data, facilitating the selection and optimization of biomaterials for cardiac applications. Young’s modulus, also referred to as the elastic modulus, measures a material’s stiffness by quantifying its resistance to deformation under tensile stress. Conductivity refers to a material’s capacity to conduct electrical current, often expressed in Siemens per centimeter (S/cm). Quantitative data has been incorporated to provide a clear and detailed comparison of the mechanical and electrical properties of different conductive biomaterials. Metrics such as tensile strength, Young’s modulus, and electrical conductivity are presented, enabling readers to understand the relative performance of materials. For example, carbon nanotube-based composites exhibit electrical conductivity values ranging from 0.1 to 10 S/cm, while polyaniline scaffolds demonstrate a Young’s modulus comparable to soft cardiac tissues, around 0.05–0.3 MPa. Following Table 3, we delve into specific failure modes and limitations observed in studies. Mechanical mismatch remains a significant challenge, as materials with a high conductivity often lack the elasticity required to mimic native cardiac tissue. Additionally, degradation issues arise when conductive materials lose structural integrity in physiological conditions, impacting long-term functionality. Finally, biocompatibility concerns, such as cytotoxicity from poorly functionalized nanomaterials, highlight the need for further research into surface modification and material optimization.

## 4. Carbon Nanomaterial Conductive Hydrogels

Carbon-based nanomaterials, including carbon nanotubes (CNTs), graphene nanosheets, and carbon nanofibers, are gaining significant interest in cardiac engineering because of their strong mechanical, topological, and electrical properties. CNTs, with their remarkable tensile properties and ability to enhance electrical signal propagation, have been extensively studied for scaffolds aimed at promoting the synchronized contraction and alignment of cardiomyocytes. However, their potential cytotoxicity necessitates functionalization techniques to improve their biocompatibility and ensure safety. Graphene and its derivatives, such as graphene oxide (GO) and reduced graphene oxide (rGO), are similarly impactful, offering superior electrical conductivity and a large surface area that supports protein adsorption and cellular adhesion. These materials enhance the coupling of cardiomyocytes and promote effective tissue integration. Despite their promise, challenges such as their limited biodegradability and potential toxicity remain barriers to clinical application. Nevertheless, the extensive body of supporting literature highlights their transformative potential in addressing damaged myocardial tissue’s critical mechanical and electrical deficiencies, paving the way for more effective cardiac repair strategies [139,140,141]. These nanomaterials are commonly combined with non-conductive hydrogels to form conductive matrices tailored for cardiac applications. CNT-based hydrogels, for example, can mimic the anisotropic structure of cardiac tissue, enhancing cardiomyocyte connectivity and function. When incorporated into methacrylate-based gelatin, CNTs create hydrogels that support synchronized beating in cardiomyocytes, replicating cardiac tissue properties. Additionally, CNTs integrated with chitosan hydrogels improve mechanical strength, functioning as nano-bridges for cardiomyocyte coupling.

Graphene-based nanosheets, such as graphene oxide (GO) and reduced graphene oxide (rGO), offer similar conductive benefits. For instance, GelMA hydrogels with rGO provide conductivity close to the natural myocardium, supporting cardiac repair and cellular function. Carbon nanofibers combined with alginate–gelatin hydrogels also enhance cell proliferation due to their mechanical strength, creating a conducive environment that supports cardiac tissue engineering [142,143,144,145,146].

Overall, CPs and carbon nanomaterials hold promise for cardiac tissue engineering, with further research needed to address challenges in biocompatibility, mechanical flexibility, and electrical stability.

## 5. Advances in Biomaterial Fabrication for Cardiovascular Engineering

In cardiac tissue engineering, various fabrication techniques are employed to create scaffolds that replicate the heart’s complex structure and function. Each method offers distinct advantages and presents specific challenges. The following Table 4 summarizes key fabrication techniques, highlighting their benefits, limitations, and applications within cardiac tissue engineering.

### 5.1. The Impact of Fabrication Parameters on Biomaterial Properties

The selection of fabrication parameters is crucial in defining the structural and functional attributes of biomaterials utilized in cardiac tissue engineering. The processing temperature significantly influences polymer crystallinity, affecting the scaffold’s mechanical strength and degradation rates. The choice of solvent impacts scaffold homogeneity and porosity, which are essential for effective cell infiltration and nutrient diffusion. In electrospinning, polymer concentration determines fiber diameter and scaffold morphology, directly influencing the replication of the extracellular matrix (ECM). Scaffold architecture, encompassing pore size, alignment, and anisotropy, plays a pivotal role in enhancing cell attachment, proliferation, and the propagation of electrical signals [16]. Optimizing these parameters enables the tailoring of scaffolds to closely mimic native cardiac tissue, thereby improving integration and functionality. This comprehensive focus on fabrication parameters underscores the intricate relationship between fabrication techniques and the resultant properties of biomaterials, offering valuable insights for the advancement of cardiac tissue engineering.

### 5.2. Additive Manufacturing for Biomedical Applications: 3D Printing and Bioprinting

**Figure 2:**
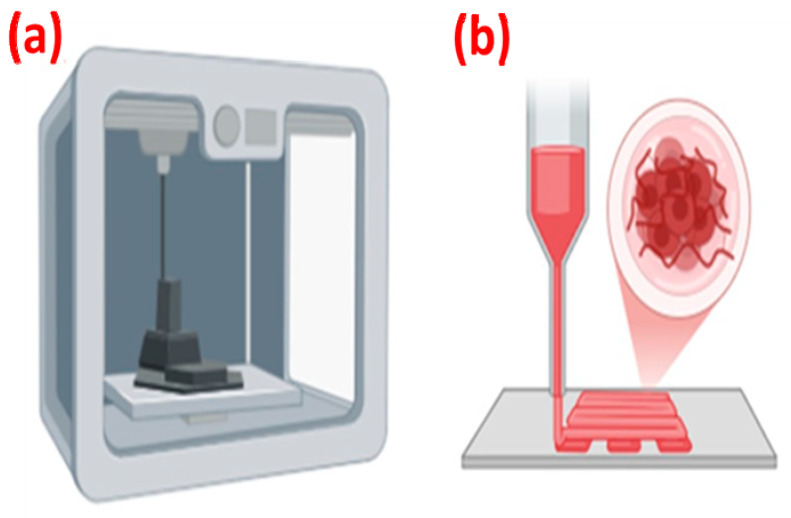
Schematic diagram of (**a**) 3D printing and (**b**) bioprinting.

Three-dimensional (3D) printing is an advanced additive manufacturing technique that enables the layer-by-layer creation of highly customized scaffolds guided by computer-aided designs. In cardiovascular applications, this technology is often used to produce patient-specific heart models, facilitating surgical planning, educational purposes, and anatomical research. When biopolymers, cells, or other biomaterials are used as printing materials, the process is called bioprinting. Bioprinting allows for the precise deposition of multiple biomaterials in a single structure, which makes it ideal for recreating the intricate structures and varied mechanical properties of tissues. Figure 2 shows a schematic diagram of 3D printing and bioprinting. The most commonly used bioprinting methods are extrusion-based inkjet and stereolithography techniques [151,152].

Bioprinting is extensively used in cardiovascular tissue engineering (CTVE) due to its capability to create complex 3D scaffolds and tissue constructs, making it valuable for regenerative therapies as well as for 3D cultures in drug testing [151,153,154]. Bioprinted scaffolds support various cardiac cells, such as cardiomyocytes (CMs), promoting a biomimetic tissue environment that enhances cell survival and function. Additionally, bioinks containing cells can be printed to produce artificial tissues directly within the scaffold. The key advantage of 3D cell bioprinting lies in its ability to accurately position cells and macromolecules in a controlled spatial arrangement. This precision is particularly valuable for creating tissue structures for implantation, including cardiac patches, heart valves, and coronary grafts [155]. However, fully functional bioprinted cardiovascular tissues with native tissue properties are not yet feasible for clinical use, signaling the need for further research in this field [152].

Furthermore, bioprinted models offer a promising alternative for drug testing and disease modeling, addressing limitations in traditional vivo and 2D models that often fall short in predicting human drug responses [155]. A continued investment in bioprinting research could pave the way for more reliable, high-throughput drug screening platforms and potentially functional tissue replacements in clinical applications. 

### 5.3. 3D Patterning Techniques

Micropatterning and microstamping are essential techniques in tissue engineering for the precise creation of complex tissue arrays. Microstamping works by modifying surfaces to attach proteins that direct cell migration and adhesion along specific patterns [156,157,158,159,160]. This control over cell interactions is crucial in 3D tissue constructs, as improper regulation often contributes to disease in multicellular tissues, particularly within cardiac and connective tissues [158,161]. These techniques are ideal for producing large-scale engineered tissues, offering high reproducibility and consistency in cellular patterns to enhance cell-to-cell communication [156,162,163]. With resolutions spanning from submicron to several centimeters, micropatterning and microstamping generate detailed patterns with exceptional fidelity [160,163,164]. These methods have effectively created cardiovascular tissue models, optimizing cell arrangement, extracellular matrix properties, and small molecule distribution for proper tissue function [165,166,167,168,169]. Integrating multiple cell types within a single structure has been demonstrated to enhance tissue maturation and promote functional development [169].

Recent innovations in patterning technology have enabled the use of biocompatible surfaces, by passing the toxic chemicals traditionally found in lithography processes [170,171]. Methods like reactive ion etching, light-activated crosslinkers, enzymatic processes, and charge-based adhesion are being employed to fabricate both rigid and flexible surfaces for cellular applications, driving progress in tissue engineering for drug discovery [158,159,165]. Mask-based photolithography, which transfers patterns onto a material through light exposure, has been utilized in forming capillary networks in vitro. In one example, endothelial cells were cultured on poly(ethylene glycol) hydrogel substrates designed via photolithography, which successfully promoted vascular network formation. When implanted in mice, these engineered networks allowed for blood cell infiltration, indicating an effective integration and perfusion [159,160]. In cardiac tissue engineering, patterned scaffolds that encourage cellular alignment are essential, as they provide a biomimetic environment that enhances cellular biomechanics [161,162]. Methods like micromolding and microablation, combined with mask-based lithography, have been used to create anisotropic surface patterns in elastomeric scaffolds, guiding the formation of aligned muscle tissues [171]. In another study, micropatterning was employed to align rat ventricular cardiomyocytes in a pattern that mirrored the structure of murine ventricular tissue, effectively replicating the native arrangement through high-resolution imaging [168].

A new cell patterning approach, bioacoustic wave patterning, has emerged for creating 3D cardiac tissue assemblies. This technique uses Faraday waves to organize cells into dense clusters in specific patterns [172,173,174]. By applying wave activation, cells suspended in a pre-polymerized hydrogel migrate from regions of a higher force to those with a lower force, leading to the formation of organized 3D structures. The hydrogel is then crosslinked, either by light or chemical methods, to stabilize the pattern [173]. Compared to other methods, wave patterning is both rapid and capable of generating consistent, repeatable patterns across large surfaces [175]. This technique has shown promise in large-scale cardiac tissue manufacturing, enabling high-throughput drug testing. For instance, human-induced pluripotent stem cell-derived cardiomyocytes were recently assembled into 3D constructs with a density similar to natural myocardium (10^8^ cells/mL) using Faraday wave patterning.

### 5.4. Textile Manufacturing

Textile fabrication methods have recently gained attention as effective approaches for manufacturing structures in cardiovascular tissue engineering (CTVE). By combining patterning techniques with additive manufacturing principles, methods like knitting, sewing, weaving, and braiding enable the creation of 3D networked structures. Among these, weaving offers the greatest design flexibility due to its anisotropic properties and interwoven connections, allowing for the production of durable structures. Textile-based implants, as seen in heart valve engineering, can be customized to mimic both the structure and function of human tissues. For example, Baaijens and his team developed a weft-knitted polycaprolactone (PCL) patch containing extracellular matrix (ECM) proteins, such as fibrinogen and thrombin, to achieve gelation. This patch demonstrated excellent durability in cyclic testing, enduring over a million cycles without failure. While this 2006 study marked a significant advancement, it faced challenges, particularly with the leakage of gelatin from the woven patch. To optimize complex architectures for cardiovascular tissue engineering (CTVE), further improvements in modeling, mechanical property tuning, and biomaterial processing are necessary [176,177,178].

### 5.5. Electrospinning Technique

**Figure 3:**
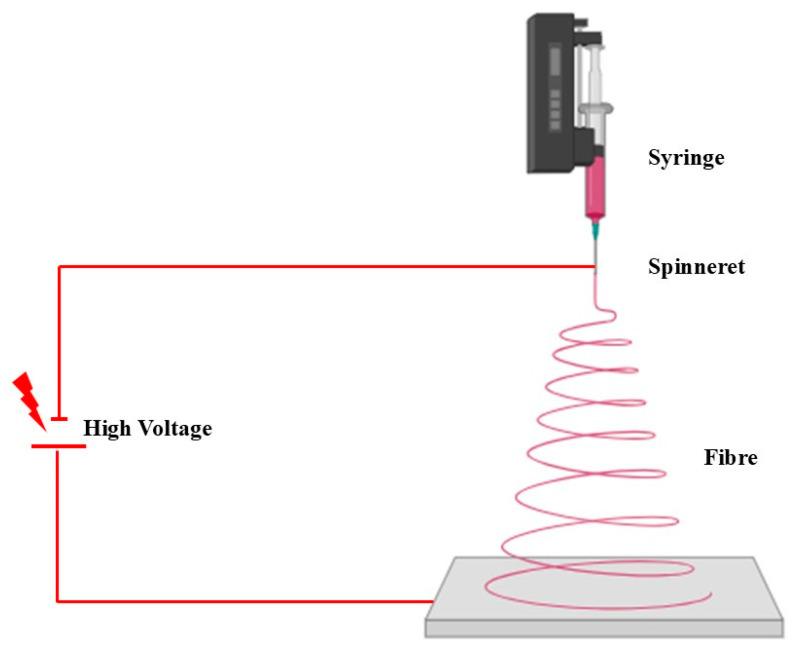
Diagrammatic illustration of an electrospinning apparatus.

Electrospinning is a fabrication method frequently used alongside additive manufacturing techniques, particularly in cardiovascular tissue engineering (CTVE). One of the key objectives in CTVE is to create graft materials that remain effective over time, integrate with living tissue, and replicate the properties of native organs. Electrospinning is a common technique used to fabricate large quantities of uniform fibers at the nanometer and micrometer scale. A polymer solution is loaded into a syringe pump, and a high voltage is applied to a conductive needle, causing the extrusion of fibers or microbeads. These fibers are collected on a grounded metal mandrel. By adjusting various process parameters, the alignment and diameter of the electrospun fibers, which range from 100 nm to 5 μm, can be precisely controlled. Figure 3 illustrates a schematic representation of an electrospinning apparatus. The syringe pump controls the rate at which the polymer solution is dispensed, enabling the precise fiber formation [179,180,181].

This method is particularly useful for creating bioresorbable grafts that can remodel in situ and closely replicate the size and structure of the extracellular matrix (ECM). As a result, electrospinning has become a widely adopted technique for producing scaffolds in various tissue engineering applications, including tendon/ligament regeneration, bone, nerve, and vascular regeneration [181,182,183].

Biodegradable scaffolds seeded with cells are commonly employed in cardiovascular tissue engineering to provide an extracellular matrix (ECM) that facilitates graft integration. For instance, Dacron vascular grafts seeded with endothelial cells (ECs) have been shown to support endothelialization and vascularization. ECs in electrospun vascular constructs have also been found to reduce thrombotic occlusions. Additionally, the combination of ECs and smooth muscle cells (SMCs) has been used to promote blood vessel formation. When seeded onto multilayer electrospun PCL-PLA scaffolds, these cells exhibited an enhanced adhesion, proliferation, and penetration [184,185,186,187,188].

Alternatively, cells can be attracted to the scaffold after implantation, where they proliferate and produce the extracellular matrix (ECM). For successful graft integration, it is crucial to thoroughly analyze the properties of the biomaterials. For example, one study showed that the composition and alignment of scaffolds affected the functionality of cardiac myocytes (CMs) in electrospun myocardial grafts. When cultured on anisotropically aligned fibers, CMs formed myocardial-like sarcomeric structures and exhibited a better performance on electrospun scaffolds blended with PGS compared to those made of gelatin. The ability to precisely tailor electrospun fibers at both the nanoscale and microscale makes this method particularly beneficial for cardiovascular tissue engineering [186,187,188].

### 5.6. Self-Assembled Biomaterial Structures

Self-assembly is a crucial process in higher organisms, playing a key role in organ growth and regeneration. It involves the spontaneous organization of individual components into complex structures with minimal external intervention. In biomaterials, self-assembly depends on biorecognition, which is the ability of biological systems to recognize and respond to specific chemical signals. This mechanism allows for the creation of structures across various scales, ranging from nanometers to macroscopic sizes. Hydrogels, a type of biomaterial, are gaining increasing attention in tissue engineering due to their self-assembling capabilities. These hydrogels provide a high-throughput, consistent, and reproducible approach for producing large quantities of biomaterials with customized properties [189,190,191,192].

A current advancement in self-assembled hydrogels involves the use of genetically engineered polymers. In this method, the polymer building blocks are encoded in genes and produced by bacteria, effectively serving as biofactories. This approach offers scalability and consistency, allowing for the creation of “smart” polymer variants that can respond to changes in pH and temperature, control cell adhesion, adjust elasticity, and maintain a precise structure, all of which are dictated by the sequence of the building blocks [193,194,195,196,197].

An emerging field in tissue engineering involves hybrid self-assembled hydrogels, which integrate two or more distinct components, such as a polymer backbone and biologically active molecules. These hybrid materials offer enhanced functionality, enabling them to perform complex tasks like controlled drug release or enzyme activation. Self-assembled hydrogels have been successfully developed as cardiac patches for treating myocardial infarction (MI). These bioengineered tissues promote significant cell proliferation, neovascularization, and the formation of intercellular junctions. In vivo applications of these cardiac patches have shown improvements in heart function by reducing left ventricular remodeling after MI, minimizing fibrosis, and preserving wall thickness, leading to a better overall cardiac health [198,199,200,201,202]. The process of studying self-assembled hydrogels through the continuous innovation of modified protein polymers has continued in the past several decades. Genes encode polymer building blocks, which bacteria produce through biological synthesis as biofactories. The production of environmental-friendly premium quality polymers requires automated genetic engineering technology. The ordered position of elastin-like polypeptides combined with silk-inspired proteins decides if elastic responses will occur or if preservation activation signaling takes place within this material. The material system contains all of the essential requirements for cardiac tissue development since it enables the healing protocols necessary for myocardial tissue restoration. End-to-end tissue engineering studies heavily depend on the development of hydrogels derived from biomolecular polymer combinations in laboratories. Multicomponent systems obtain healing properties due to drug delivery systems that merge detection platforms with medication administration strategies. A scientific research team designed heart patches using synthetic compounds for treating myocardial infarction (MI) patients. Through its medical engineering process, the construction develops new vascular connections that establish critical links to the heart. Using this treatment on cardiac cells allows organisms to obtain several therapeutic advantages:

∘ Reduced left ventricular remodeling after MI
∘ Minimized fibrosis
∘ Preserved myocardial wall thickness
∘ Enhanced cardiac contractility and function

Hydrogels that assemble themselves will help heart healing applications in future regenerative medicine therapy, according to medical experts.

### 5.7. Shape Memory Structures

Advanced materials known as shape memory materials exhibit two activating features through temporary deformations, which can be triggered by temperature, light, and magnetic fields. Medical practitioners use shape memory polymers (SMPs) derived from polymers and metals in their research due to their cost-effectiveness and suitable medical properties for biomedical applications. Thermoset or thermoplastic SMPs are programmed using two methods: heating the material past its transition temperature (T_trans) or permanently deforming it below T_trans. After undergoing deformation, these materials return to their original state when exposed to the appropriate stimulus [203,204,205,206]. SMPs can be designed to produce variously sized shapes with customizable functional properties. In the biomedical field, SMP variants such as polyurethane (PU), polycaprolactone (PCL), polylacticacid (PLA), and epoxy-based thermosets are widely used due to their unique advantages in medical applications. PCL-based SMPs exhibit biodegradability, making them suitable for tissue engineering structures, while PU-based SMPs possess excellent elasticity and durability, making them ideal for cardiovascular stents and catheters. PLA is preferred for its strength and stiffness, while epoxy-based thermosets are valued for their shape recovery and stability, making them optimal materials for self-expanding medical devices. Current 3D printing technologies allow SMPs to be incorporated into multi-material systems, enabling the precise fabrication of functional components. In 3D bioprinting applications, PCL and PLA biodegradable polymers serve as essential components for SMP scaffolds, providing structural support while enabling shape transformations that enhance cell-to-cell interactions and promote tissue development. Additionally, PU SMPs fabricated through stereolithography (SLA) are utilized in biomedical applications, including drug delivery systems and active medical implant structures. The primary medical application of SMPs involves the development of minimally invasive surgical devices, including arterial catheters, ventricular cardiac leads, and stents [207,208,209,210]. Research in cardiology is focused on leveraging SMPs for cardiac stents and catheter systems. These medical devices benefit from SMPs precise structural control mechanisms and thermally trigger deployment properties at human body temperatures. However, SMP applications face two significant challenges: the difficulty of creating intricate geometric shapes due to self-interference and the need to ensure the long-term biocompatibility of specific SMP materials. Moreover, healthcare applications of SMPs are constrained by complex manufacturing requirements, which must preserve their macro properties during functionalization processes. Despite these challenges, advancements in shape memory materials continue to drive progress toward clinical applications, with a promising potential for future medical innovations [210,211,212].

### 5.8. Conventional Casting Methods

Traditional 3D casting (molding) techniques have utilized various hydrogel biomaterials, such as collagen, fibrin, and gelatin, to fabricate scaffold structures for cardiovascular tissue engineering applications [174,196,197,212,213,214,215]. For instance, type I collagen gels were poured into molds with specific shapes, such as 24-well culture plates for mouse studies and 6-well plates for pig studies. The resulting hydrogel was then compressed to form a cardiac patch that mimicked embryonic tissue [196,197,213,216]. The effect of applying an epicardial patch on heart function was evaluated in both mouse and pig models of myocardial infarction (MI) [215,217,218,219]. In both animal models, the implantation of the engineered collagen patch loaded with cardiogenic compounds led to notable improvements in myocardial structure and function [196]. In both animal models, the implantation of the engineered collagen patch loaded with cardiogenic compounds led to notable improvements in myocardial structure and function [220,221].

Solvent casting is another technique used in tissue scaffold engineering, where biocompatible, biodegradable materials are shaped using water-soluble particulates in molds. The solvent evaporates, and the particulates are washed away, leaving a porous scaffold structure behind. This method has been employed to create biosynthetic heart valves using biomaterials like PLA and PGA. Once the scaffolds are fabricated, cells can be seeded onto them to proliferate, differentiate, and remodel the matrix, promoting a neo-tissue formation. The presence of interconnected pores in the scaffold is essential for cell viability and function, as they facilitate the exchange of nutrients and waste [222,223].

A significant concern with solvent casting is the potential for toxic solvent residues remaining within the scaffolds. This issue can be mitigated by thoroughly lyophilizing (vacuum drying) the scaffolds to remove any residual toxic substances. Another approach to scaffold fabrication involves combining solvent casting with particle leaching techniques. For instance, Johnson et al. utilized poly(vinyl pyrrolidone) (PVP) and poly(vinyl alcohol) (PVA) as dispersing agents in two solvent-casting and particle-leaching methods to create cylindrical porous scaffolds. Their study demonstrated that adjusting the porogen type resulted in scaffolds with varying pore sizes and interconnectivity, likely due to the different adsorption rates of PVP and PVA. Furthermore, the scaffolds showed distinct responses in terms of cell attachment, viability, and spreading when cultured with human coronary artery smooth muscle cells [223].

Freeze drying is another sublimation-based technique for creating porous scaffolds. This process begins by dissolving a polymer in a solvent to form a solution, which is then frozen in a mold. Through vacuum lyophilization, the solvent is removed, resulting in a scaffold with a highly porous structure. Biopolymers used in freezing drying include silk, PGA, PLLA, PLGA, and their blends. One of the key advantages of this approach is its relatively simple fabrication process, which avoids the need for high temperatures or separate leaching steps. However, it also has some limitations, such as challenges in incorporating cells into the polymer solution due to the harsh fabrication conditions, smaller pore sizes, and longer processing times [224,225].

### 5.9. Current Challenges and Limitations

Despite significant advancements, conductive biomaterials for cardiac tissue engineering face numerous challenges that limit their clinical application. Biocompatibility and immune response issues remain critical concerns, as some conductive materials, particularly carbon-based nanomaterials like CNTs and graphene, may elicit adverse cellular or systemic reactions without proper functionalization. Achieving an optimal integration of the mechanical and electrical properties is another hurdle, as many materials with excellent electrical conductivity lack the mechanical strength required to mimic native cardiac tissue. Additionally, fabrication techniques, such as electrospinning and 3D printing, while innovative, often face limitations in scalability and consistency, making them challenging for large-scale production. Furthermore, the environmental and ethical considerations of material sourcing, including the extraction and processing of nanomaterials, raise sustainability concerns. Addressing these limitations through multidisciplinary research and innovation is essential to bridge the gap between laboratory success and clinical translation.

## 6. Conclusions

The combination of electrical conductivity, tunable mechanical properties, and bioactive molecule delivery makes these composites highly suited for developing tissue scaffolds that foster cell growth and maintain structural integrity. Recent research highlights their promise in regenerating various tissues, including bone, skin, nerves, and heart. Nonetheless, considerable progress is needed to transition these materials from proof-of-concept stages to viable clinical therapies. Most studies have focused on establishing biofunctionality and therapeutic potential through in vitro and animal models. Extensive preclinical testing for biocompatibility and toxicity is necessary before advancing to human trials. Additionally, long-term in vivo studies are essential to confirm that these materials degrade safely and effectively aid tissue repair. Developing scalable manufacturing techniques and stringent quality control processes is also crucial for producing these composites consistently and affordably for clinical use. Additionally, scalable manufacturing methods and robust quality control processes need to be developed to produce these composites consistently and cost-effectively for clinical applications.

The findings suggest that conductive biomaterials, with or without external electrical stimulation, can enhance cardiac cell function, contributing to the restoration of myocardial electromechanical integrity and promoting cardiac repair and regeneration. However, in vivo studies involving conductive materials are still limited, and more research is needed to confirm their biocompatibility, degradation rates, and potential for immunogenicity. Future research will likely focus on understanding the molecular mechanisms behind cellular responses to these materials while also evaluating the genotoxicity and cytotoxicity of electroactive biomaterials. The results of these studies will pave the way for the use of conductive materials in clinical cardiac applications. Scaffolds for cardiac tissue engineering should closely replicate the myocardial extracellular matrix (ECM), with mechanical, structural, and electrical properties that resemble native myocardium, to create an optimal environment for cell adhesion, growth, and proliferation. Researchers are increasingly exploring a variety of conductive biomaterials, including those based on carbon, metals, and electroactive polymers, to enhance the electrical interactions within scaffolds and improve outcomes in cardiac tissue engineering.

## Figures and Tables

**Table 1:** Natural polymers in cardiac tissue engineering.

Scaffold	Polymer	Applications in Cardiac Tissue Engineering	Advantages	Limitations	References
Protein-based materials	Fibrin	Injectable scaffolds and cell carriers for tissue engineering	Controlled degradation rate, non-toxic degradation products, high cell seeding efficiency, uniform cell distribution, and enhanced cellular interaction	Weak mechanical properties, prone to shrinkage and contraction	[29,30]
	Collagen	Scaffolds for differentiation into cardiac progenitor cells and cardiac patches	Low immunogenicity, porous structure, excellent permeability, biocompatibility, and biodegradability	Poor mechanical properties, limited structural stability, and low electrical conductivity	[31,32]
Natural polymers	Gelatin	Delivery vehicles for stem cells and biomolecules; scaffolds for myocardial repair	Excellent cell response features	Weak mechanical properties, reduced stability, low melting point, lack of 3D structural integrity, rapid dissolution in water	[33,34,35,36]
	Matrigel	Supporting cardiomyocyte differentiation and vascularization	Cytocompatibility, intrinsic stiffness	Derived from murine sarcoma cells, limiting clinical application	[37,38]
Polysaccharides	Alginate	Injectable gels for cardiac repair and drug delivery systems	Non-toxic, biocompatible, and biodegradable	Time-consuming processing, rapid disintegration in bodily fluids, low drug entrapment efficiency, quick drug release	[39,40,41]
	Chitosan	Scaffolds with enhanced electrical properties and drug delivery systems	Low immunogenicity, processable into various structures, biocompatible, good mucoadhesive properties	Weak mechanical properties, limited electrical conductivity	[42,43,44]

**Table 2:** Synthetic polymers and their properties in cardiac tissue engineering.

Material Type	Material	Mechanical Properties	Electrical Properties	Biological Properties	Applications	References
Biodegradable Polymers	Polylactic acid (PLA)	High strength, good processability	Non-conductive	Biocompatible, biodegradable	Scaffolds for cardiac patches, drug delivery systems	[71]
	Polycaprolactone (PCL)	Flexible, strong	Non-conductive	Biocompatible, slow degradation	Long-term cardiac scaffolds, slow-release drug delivery systems, developments in biomaterials for regenerative medicine and cardiac tissue engineering	[72]
Conductive Polymers	Polypyrrole (PPy)	Moderate mechanical properties	Conductive (0.1–100 S/cm)	Supports cell adhesion and proliferation	Conductive cardiac scaffolds, electrically active tissue engineering	[71]
Composites	Carbon nanotubes (CNTs)	Excellent mechanical strength	High conductivity	Biocompatible with proper functionalization	Reinforced scaffolds, electrical signal restoration	[73]
Graphene-Based Materials	High strength and flexibility	Highly conductive	Biocompatible in low doses, can enhance cell behavior	Scaffolds for enhancing electrical conductivity in myocardial tissue	Biomaterials engineering to guide heart cells in developing mature cardiac tissue	[71]
Hydrogels	PEG-based hydrogels	Adjustable mechanical properties via crosslinking	Non-conductive unless modified	Biocompatible, hydrophilic	Injectable scaffolds, provides supports for vascularization	[74]
Hybrid Hydrogels (e.g., GelMA-CNT)	Tunable properties based on composition	Conductive when CNT or PPy integrated	Supports cell proliferation and vascularization	Multifunctional scaffolds combining mechanical, electrical, and biological properties for cardiac repair	Biomaterials engineering to guide heart cells in developing mature cardiac tissue	[71]

**Table 3:** Comparative properties of the conductive biomaterials.

Type	Material	Electrical Conductivity (S/cm)	Young’s Modulus (MPa)	Biocompatibility	Key Limitations	References
Inorganic	Carbon Nanotubes	0.1–10	1–10	High (with functionalization)	Potential cytotoxicity	[135]
	Graphene Oxide	10–100	0.01–0.1	High	Potential degradation issues	[136]
Organic	Polypyrrole	0.05–0.1	0.1–0.5	Moderate	Mechanical brittleness	[137]
	Polyaniline	0.01–5	0.05–0.3	High (post-modification)	Poor processability	[138]

**Table 4:** Fabrication techniques in cardiac tissue engineering.

Technique	Advantages	Disadvantages	Examples in Cardiac Tissue Engineering	Reference
Electrospinning	Produces nanoscale fibers with a high surface area, tunable porosity, and biomimetic structure	Limited control over scaffold thickness and cell distribution	Fabrication of conductive scaffolds using polyaniline or CNTs for enhanced conductivity	[16]
3D Printing	High precision, customization of complex geometries, integration of multiple materials	Time-intensive requires biocompatible and printable conductive inks	Printing of conductive cardiac patches using graphene or polypyrrole composites	[146]
Solvent Casting	Simple, cost-effective, and scalable	Limited control over microarchitecture, uneven distribution of conductive materials	Development of thin conductive films incorporating carbon nanotubes for cardiac scaffolds	[147]
Melt Electrospinning	Combines the precision of 3D printing with electrospinning-like properties	Limited material compatibility, high processing temperatures	Fabrication of aligned fiber scaffolds with conductive biomaterials for cardiac alignment	[148]
Freeze-Drying	Generates highly porous, lightweight structures suitable for cell infiltration	Fragile structures, challenging integration of conductive materials	Preparation of conductive hydrogels infused with graphene oxide for myocardial repair	[149]
Spin Coating	- Produces uniform thin films - Rapid and scalable process - Allows for multilayer coatings	- Limited to simple geometries - Potential for an uneven coating on complex surfaces	Application of conductive polymer coatings on cardiac implants. Development of bioactive surfaces enhancing cell adhesion	[150]
